# Alpha-lipoic acid does not improve olfactory training results in olfactory loss due to COVID-19: a double-blind randomized trial

**DOI:** 10.1016/j.bjorl.2023.101356

**Published:** 2023-10-30

**Authors:** Lorena Pinheiro Figueiredo, Paulo Victor dos Santos Lima Paim, Thiago Cerqueira-Silva, Carolina Cincurá Barreto, Marcus Miranda Lessa

**Affiliations:** a Universidade Federal da Bahia (UFBA), Programa de Pós-Graduação em Ciências da Saúde, Salvador, BA, Brazil; b Universidade Federal da Bahia (UFBA), Faculdade de Medicina, Salvador, BA, Brazil; c Instituto Gonçalo Moniz, Fiocruz, Salvador, BA, Brazil; d Universidade Federal da Bahia (UFBA), Hospital Universitário Professor Edgard Santos (HUPES), Serviço de Otorrinolaringologia, Salvador, BA, Brazil

**Keywords:** COVID-19, Alpha-lipoic acid, Olfactory dysfunction, Olfactory test, Post acute COVID-19 syndrome

## Abstract

**Objectives:**

Olfactory loss is a recognized long-term dysfunction after Coronavirus Disease 2019 (COVID-19) infection. This investigation aimed to assess the effect of alpha-lipoic acid as an adjuvant treatment of olfactory training on the improvement of smell loss in post-COVID-19 patients.

**Methods:**

This randomized controlled trial included 128 adult outpatients who had persistent smell loss for more than 3-months after COVID-19 infection. The participants were randomly allocated into two groups: the intervention treatment group, which received alpha-lipoic acid associated to olfactory training, and comparison treatment group, which received placebo pills associated to olfactory training. The participants were followed-up for 12-weeks. Olfactory dysfunction was assessed in terms of Visual Analog Scale (VAS), and the Connecticut Chemosensory Clinical Research Center (CCCRC) test for the Brazilian population.

**Results:**

A total of 100 participants completed the follow-up period and were analyzed in this study. Both groups have improved CCCRC score (*p* = 0.000), olfactory threshold (*p* = 0.000), identification score (*p* = 0.000) and VAS score (*p* = 0.000) after 12-weeks follow-up. No significant differences were determined between the intervention and comparison treatment groups in CCCRC score (*p* = 0.63), olfactory threshold (*p* = 0.50), identification score (*p* = 0.96) and VAS score (*p* = 0.97). In all these criteria, comparison treatment group went slightly worse. At the endpoint of the study, the frequency of anosmia reduced to 2% in the intervention treatment group and to 7.8% in the comparison treatment group. Also, 16.8% of the intervention group’ subjects, and 15.7% of comparison treatment group’s patients reached normosmia.

**Conclusions:**

Overall, there was a strongly significant difference in olfactory function between baseline and endpoint for both groups. However, based on the lack of significant difference between the intervention treatment and the comparison treatment groups in terms of olfactory changes, our study appoints that the alpha-lipoic acid is not better than olfactory training alone to treat olfactory loss after COVID-19.

**Level of evidence:**

Level 2.

## Highlights

• Alpha-lipoic acid is not better than olfactory training alone after COVID-19.
• We found a strongly difference in baseline and endline olfaction in both groups.
• This is the first trial comparing alpha-lipoic acid as an adjuvant treatment.

## Introduction

At the time of writing (July 9th, 2023), there have been more than 676 million confirmed cases of COVID-19 worldwide.^1^ These, 37.69 million have happened in Brazil.^2^

Loss of smell is a very common symptom in COVID-19. It affected between 64.76% and 67.49% patients included in a cohort tracking of self-reported symptoms in United States and United Kingdom.^3^ That study published in the early pandemic also showed that loss of smell was a high predictive symptom of COVID-19.

One year later, a Brazilian group confirmed the high prevalence of olfactory disorders in patients with early COVID-19. According to Brandão Neto et al., 65.1% of COVID-19 patients had smell complaints.^4^ In their prospective observational study, 44.7% (95% CI 36.5%‒53%) had partial recovery and 1.4% (95% CI 0%‒3.3%), presented no recovery of smell loss. Considering the high incidence of COVID-19 until here, there will be a huge number of patients with smell loss needing care, treatment, and rehabilitation.

Since the pandemic started, the knowledge about loss of smell due to COVID-19 has been increased. Before pandemic, there was some therapies used to recover loss of smell. Alpha-lipoic acid^5^ and olfactory training^6^ showed encouraging results.

Alpha-lipoic acid (ALA) is a fatty acid that presents antioxidative effects, and neuronal regenerative capabilities. Accordingly, Hummel et al. showed that 61% of patients with smell loss after upper respiratory viral infection has improved olfactory function using 600 mg/day of ALA, for an average period of 4.5-months.^5^

In a later study, the same work group has shown an increase of olfactory function in patients who performed a 12-week olfactory training protocol twice a day to four intense odorants (phenyl ethyl alcohol: rose, eucalyptol: eucalyptus, citronellal: lemon and eugenol: cloves). That study included postinfectious, post traumatic and idiopathic smell disorders’ patients, and evaluated Sniff and Stick’s test results.^6^

There is enough literature regarding olfactory training to recover olfactory loss due to COVID-19. As an example, Pires et al. showed significant olfactory improvement in patients with smell loss due to COVID-19 receiving OT with 4 or 8 essences after 4 weeks.^7^ Nevertheless, there is not enough literature for alpha-lipoic acid use in this case.

The aim of this study is to assess the effect of alpha-lipoic acid as an adjuvant of Olfactory Training (OT) on the improvement of smell loss in post-COVID-19 patients.

## Methods

### Study design

This is a double-blind, two-arm, randomized, placebo-controlled trial to assess the effect of ALA as an adjuvant of olfactory training on the improvement of olfactory loss in COVID-19 long-term outpatients. The study protocol was approved by the Ethics Committee of Federal University of Bahia (Ethical code: 4.562.570). All patients included in the study assigned informed consent before participation. Additionally, our trial study was approved in the Brazilian registry of clinical trials (U1111-1267-0901).

### Patients

The inclusion criteria were: 1) adult between 18 and 65 years of age; 2) previous SARS-CoV-2 infection confirmed after a positive reverse transcription-polymerase chain reaction or antigen test; 3) olfactive disorder lasting more than three months. Furthermore, the smell loss had to be confirmed by Connecticut Chemosensory Clinical Research Center (CCCRC) test score <6.0.

We excluded: 1) patients with a history of previous olfactory dysfunction, trauma, surgery, or radiotherapy in the oral and nasal cavities; 2) patients with chronic rhinosinusitis, psychiatric or neurological diseases, recent use of corticosteroids earlier than 30 days or current use of alpha-lipoic acid, omega 3, vitamin A, zinc or pentoxifylline.

### Data collect

The demographic characteristics, medical history, symptoms, duration of olfactory dysfunction and outcomes data were collected during the first visit. Before being examined, patients were asked about their subjective olfactory score using a Visual Analogic Scale (VAS) from 0 to 10.

### Study setting and interventions

The patients included in the study were randomly divided into two groups using a computer-generated random number table. The patients and the physician were both completely blinded, and the assignment and the randomization were performed by a third person.

The intervention treatment group received one bottle containing 180 pills of alpha-lipoic acid 300 mg/pill and the comparison treatment group received one identical bottle including 180 pills of placebo (starch) 300 mg/pill. The patients should take 01 pill twice a day, for 12-weeks, followed by Hummel et al. previous study.^5^

Furthermore, both groups received an olfactory training kit with four odorants (phenyl ethyl alcohol: rose, eucalyptol: eucalyptus, citronellal: lemon and eugenol: cloves), in plastic pots labelled with the odor name (made by Clínica Olfact- Londrina/PR-Brazil). Inside, there was a cotton pad soaked with 1 mL of each scent. Patients were advised to sniff the odors twice a day for approximately 10 s each. To help therapeutic adherence, patients received a diary to take notes daily. Moreover, patients received a phone call by every 4-weeks into the treatment period to ask about the patients’ subjective olfactory function by Visual Analogic Scale, to maintain compliance to the study and to collect data about side effects.

### Olfactory function

Connecticut Chemosensory Clinical Research Center (CCCRC) test was used to assess olfactory function of all the patients.^8^ Each patient performed the test twice: in the enrollment and after 12-weeks of treatment. The test is composed of two stages: 1) butanol test to determine threshold; 2) olfactory identification test with 8 scents.

Threshold testing was performed presenting solutions of *N*-butanol in decreasing concentration in 8 steps. The strongest butanol concentration was 4%. Each other bottle (from 2 to 7) contained a subsequent 1:3 *N*-butanol dilution. Bottle 8 was filled by deionized water. Two identical squeezable bottles were presented to the patient: one containing the *N*-butanol solution, starting from the major dilution, and the other filled with deionized water. Non tested nostril was closed by a micropore tape. Patient was asked to close the eyes and smell each bottle, reporting which of the two ones smelled most. The threshold was identified when the subject gave the correct answer four times. In case of error, the next most concentrated solution was given to the patient. The threshold was quantified for each of the two nostrils with a score from 0 to 7 corresponding to less concentrated bottle that the patient was able to correctly detect. The average between values of the two nostrils expressed the overall score.

For the identification test, eight well-known Brazilian odorants were used: coffee, soap, Cinnamomum, *paçoca* (a peanut sweet), chocolate, baby powder, camphor ball and Vick VapoRub®. Each step was performed through both nasal cavities and received a score from 0 to 7.

At the end of the process, a final score was calculated, following the equation: average score obtained in the butanol test for both nostrils plus average of the discrimination score for the two nostrils/2, resulting in a score also from 0 to 7 that determined the degree of hyposmia. Normosmia was considered as a score between 6.0 and 7.0; mild hyposmia, from 5.0 to 5.75; moderate hyposmia, from 4.0 to 4.75, severe hyposmia, from 2.0 to 3.75, and anosmia, from 0 to 1.75.

After the olfactory test, patients underwent to physical examination and nasal endoscopy.

### Primary outcomes

At the end of 12-weeks, patients returned to outpatient clinic. The visual analogue scale was applied, the diary was returned, and endpoint CCCRC was performed. The primary outcomes were CCCRC and VAS score changes in both groups.

### Sample size

The sample size was determined based on the alpha error of 0.05, a power of 80% (beta error = 0.2), and a confidence level of 95%. We expected 4.4 the magnitude of the effect of ALA in the smell recovery. Thus, the calculated sample size was 64 subjects in each group.

### Statistical analysis

Statistical analysis was performed with the SPSS 25th version (IBM, Armonk, NY, USA). Qualitative variables were described using frequency (%) and compared by Chi‐Square test. The quantitative variables were checked using the Kolmogorov–Smirnov test to determine the normal distribution of mentioned data. Accordingly, the variables with or without normal distribution were reported as the mean ± standard deviation, or median and interquartile range (percentile 25–75), respectively. The Unpaired sample t‐test or Mann–Whitney test was performed to compare quantitative variables between placebo and intervention groups. Additionally, mixed Analysis of Variance (ANOVA) was used to assess differences between ad within groups. The *p*‐value <0.05 was considered significant.

## Results

**Figure 1:**
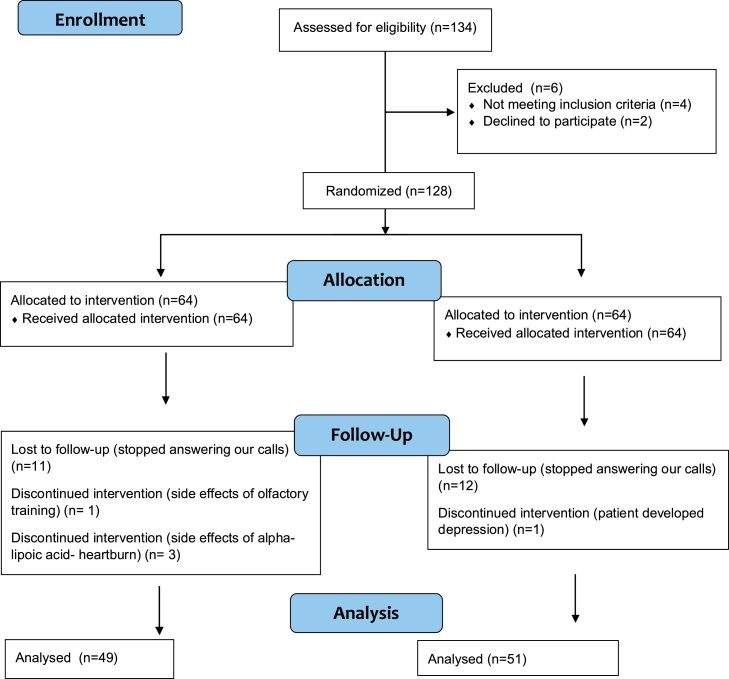
Study CONSORT flow diagram.

Between March 1st and October 30th, 2021, 134 subjects were assessed for eligibility, and 128 patients were included in the present study. The participants were randomly divided into two groups to receive olfactory training with alpha-lipoic acid 600 mg a day (intervention treatment group) or olfactory training with placebo pills 600 mg a day (comparison treatment group), both for 12-weeks. Fifteen subjects in the intervention group and thirteen subjects in the comparison treatment group were excluded through the follow-up period. Forty-nine patients were analyzed in the intervention treatment group, while 51 patients were analyzed in comparison treatment group to evaluate the recovery of olfactory dysfunction (Fig. 1).

According to Table 1, the mean age was 38.2 ± 11.3 and 39.9 ± 13.3 years in the intervention and placebo groups, respectively, with no significant difference between the two groups (*p* = 0.44). The duration of olfactory dysfunction of subjects before intervention was 8 ± 7 months in the intervention group and 7 ± 5.5 months in the placebo group (*p* = 0.39). 79.6% and 84.3% of patients were female in the intervention and placebo groups, respectively (*p* = 0.83). Side effects were noted in both groups. The most frequent were burning the stomach and heartburn in both groups (26.5% in intervention group; 7.8% in comparison treatment group).

**Table 1:** The comparison of demographics and COVID-19 clinical details between intervention and comparison groups.

Variable	Intervention (n = 49)	Comparison (n = 51)	*p*-Value
Age, mean (SD), years	38.2 ± 11.3	39.9 ± 13.3	0.44^a^

Female frequency, %	79.6	84.3	0.83^b^

Schoolling level			
<4 years, %	2.5	2.6	0.83^c^
4‒7 years, %	0	5.3	
8‒11 years, %	15	15.8	
>12 years, %	82.5	76.3	

Diagnosis method of COVID-19			
RT-PCR, %	65.3	72.5	0.15^b^
Antigen test, %	34.7	27.5	

Duration of olfactory loss [IQR], months	8 [5–12]	7 [5–10.5]	0.39^d^

COVID-19 severity			
Mild, %	93.9	96.1	0.27^c^
Moderate, %	6.1	3.9	
Severe, %	0	0	

Parosmia, %	57.1	49	0.19^b^
Dysgeusia, %	53.2	60.8	0.73^b^

Baseline CCCRC score, mean (SD)	2.7 ± 1.5	2.9 ± 1.4	0.84^a^
Olfactory thereshold, mean (SD)	2 ± 1.4	1.9 ± 1.3	0.89^a^
Identification, mean (SD)	3.4 ± 1.9	3.8 ± 1.9	0.65^a^

Baseline olfactory loss severity			
Mild hyposmia, %	8.2	9.8	0.49^b^
Moderate hyposmia, %	18.4	19.6	
Severe hyposmia, %	53.1	54.9	
Anosmia, %	20.4	15.7	

Baseline VAS score, median [IQR]	2.5 [0–5]	3 [1–5]	0.58^d^

SD, standard deviation; IQR, interquartile rage; RT-PCR, reverse transcription polymerase chain reaction; COVID-19, coronavirus disease 2019; CCCRC, Connecticut Chemosensory Clinical Research Center; VAS, Visual Analogic Scale.

a Student *t*-test.

b X² test.

c Fisher’s exact test.

d Wilcoxon–Mann–Whitney test.

Additionally, no significant differences were observed in terms of COVID-19 severity (*p* = 0.27), baseline CCCRC score (*p* = 0.84), baseline olfactory loss severity (*p* = 0.49) or baseline VAS score (*p* = 0.58) between the two groups, before treatment.

Table 2 shows the olfactory scores according to the smell test and VAS in the follow-up period. By comparing the mean scores of smell test based on CCCRC, both groups have improved CCCRC score (*p* = 0.000), olfactory threshold (*p* = 0.000), identification score (*p* = 0.000) and VAS score (*p* = 0.000) 12-weeks after treatment.

**Table 2:** Comparison of olfactory evaluation based on CCCRC test and VAS score between and within the intervention and comparison groups.

Olfactory evaluation	Baseline	Final (12 w)	*p-*Value with in group^a^	*p*-Value between groups^a^
CCCRC score, mean (SD)				
Intervention group (OT + ALA)	2.7 ± 1.5	4.6 ± 1.3	*p* < 0.001	*p* = 0.63
Comparison group (OT)	2.9 ± 1.4	4.3 ± 1.6	*p* < 0.001	

Olfactory thereshold, mean (SD)				
Intervention group (OT + ALA)	2 ± 1.4	4.2 ± 1.7	*p* < 0.001	*p* = 0.50
Comparison group (OT)	1.9 ± 1.3	3.8 ± 1.9	*p* < 0.001	

Identification, mean (SD)				
Intervention group (OT + ALA)	3.4 ± 1.9	4.9 ± 1.5	*p* < 0.001	*p* = 0.96
Comparison group (OT)	3.8 ± 1.9	4.9 ± 1.8	*p* < 0.001	

VAS score, median [IQR]				
Intervention group (OT + ALA)	2.5 [0–5]	6 [4–8]	*p* < 0.001	*p* = 0.97
Comparison group (OT)	3 [1–5]	6.5 [5–8]	*p* < 0.001	

12W, Revaluation after 12-weeks; SD, standard deviation; OT, olfactoy training; ALA, alpha-lipoic acid; bilat., bilaterally; VAS, Visual Scale.

a Mixed Design Analysis of Variance (ANOVA), and post-hoc Sidak test.

No significant differences were determined between the intervention and comparison groups in CCCRC score (*p* = 0.63). Additionally, there were no statistically significant differences between groups in mean scores concerning olfactory threshold, identification score and VAS score (*p* = 0.50, 0.96 and 0.97, respectively).

Our results revealed that the mean change of smell identification test and VAS scores were 1.65 ± 1.02 and 3.5 ± 2.3 in the intervention group, versus 1.28 ± 1.3 and 3 ± 2.8 in the comparison group, after 12-weeks, respectively. The analysis between CCCRC mean change and VAS mean change did not show statistically significant differences (*p* = 0.20 and *p* = 0.62, respectively, after Student’s test-*t*).

**Figure 2:**
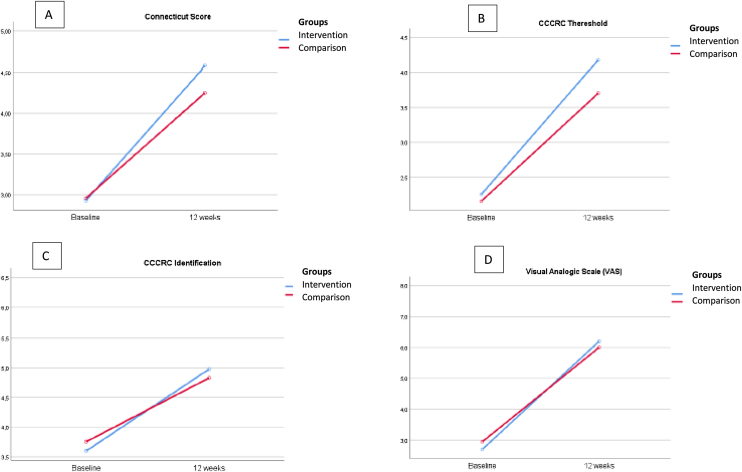
Comparison between Connecticut score, its olfactory domains and VAS in the two groups after treatment. (A) Comparison between Connecticut Chemosensory Clinical Research Center test score in two groups before and after treatment. (B) Comparison between Connecticut Chemosensory Clinical Research Center test threshold in two groups before and after treatment. (C) Comparison between Connecticut Chemosensory Clinical Research Center test identification in two groups before and after treatment. (D) Comparison between Visual Analogic Scale (VAS) in two groups before and after treatment.

Fig. 2 shows the olfactory evaluation in both groups over 12-weeks.

According to Table 3, the olfactory dysfunction such as anosmia, severe or mild hyposmia based on smell test did not show differences in baseline and 12-weeks after treatment in both groups.

**Table 3:** Evaluation of the olfactory dysfunction types such as anosmia, and severe or mild hyposmia based on smell test between two groups in the follow‐up period.

Variable	Intervention (n = 49) frequency (%)	Comparison (n = 51) frequency (%)	*p*-Value
Baseline			
Anosmia	10 (20.4%)	8 (15.7%)	
Severe hyposmia	26 (53%)	28 (54.9%)	0.49
Moderate hyposmia	9 (18.4%)	9 (19.6%)	
Mild hyposmia	4 (8.2%)	5 (9.8%)	

After 12-weeks			
Anosmia	1 (2%)	4 (7.8%)	
Severe hyposmia	15 (30.6%)	15 (29.4%)	
Moderate hyposmia	13 (26.6%)	12 (23.5%)	0.96
Mild hyposmia	12 (24.5%)	12 (23.5%)	
Normosmia	8 (16.3%)	8 (15.7%)	

**Figure 3:**
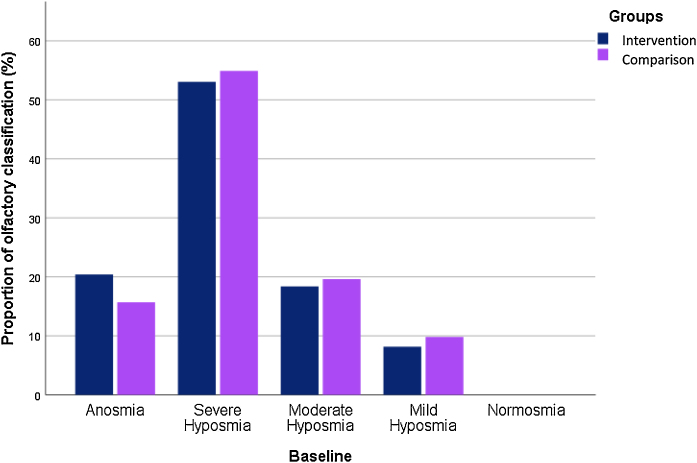
Baseline olfactory dysfunction classification in the two groups.

**Figure 4:**
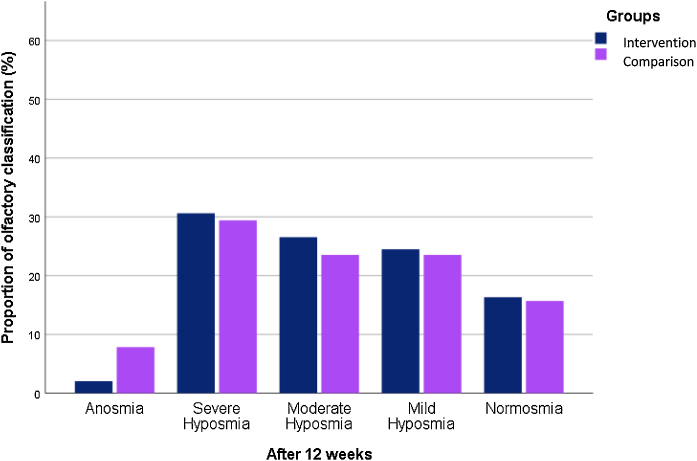
Endpoint olfactory dysfunction classification in the two groups.

Anosmia was reported at 20.4% and 15.7% in the intervention and comparison groups, respectively. At the endpoint of the study, the frequency of anosmia reduced to 2% in the case group and to 7.8% in the placebo group. 16.8% of intervention subjects and 15.7% of patients in the comparison group reached normosmia (Figure 3, Figure 4).

However, no significant differences were observed between groups regarding the severity of smell dysfunction during the follow-up period.

## Discussion

This study is the first trial assessing the effect of alpha-lipoic acid as an adjuvant of olfactory training on the improvement of smell loss in post-COVID-19 patients.

Herein, we found a strong statistically significant differences in CCCRC olfactory scores and VAS in the follow-up period in both groups. Comparing the mean scores of smell tests, both groups have improved CCCRC score (*p* < 0.001), olfactory threshold (*p* < 0.001), identification score (*p* < 0.001) and VAS score (*p* < 0.001) after 12-weeks of treatment. Also, 16.3% of intervention treatment group and 15.7% of the comparison treatment patients reached normosmia after the study.

In 2002, Hummel et al. concluded that alpha-lipoic acid (ALA) might be helpful in patients with olfactory loss after upper respiratory tract.^5^ This classical study was a non-blinded prospective trial, with only 23 patients at that time. Ever since, no other clinical trials have been done testing this drug in olfactory function.

Alpha-lipoic acid is a fatty acid that penetrates the blood–brain barrier. It is metabolized into cell to its active metabolite, Dihydro-Lipoic Acid (DHLA).^9^ Both ALA and DHLA have a determinant role in oxidative metabolism. While ALA reduces singlet oxygen, peroxynitrite, hypochlorous acid, and hydroxyl radicals, DHLA decreases superoxide and peroxyl radicals and regenerates vitamin E by elevating intracellular glutathione concentrations.^10^ Thus, both ALA and DHLA show properties to be useful in repairing oxidative lesions.

Regarding SARS-CoV-2’s physiopathology of the infection, it is important to remember that SARS-CoV adheres its Spike protein (S) to the host cell membrane through the Angiotensin-Converting Enzyme 2 (ACE-2) receptor.^11^ As a result, tissues with higher expression of this receptor are most vulnerable to the effects of SARS-CoV-2. Olfactory neuroepithelium is one of these tissues.

In the specific case of the olfactory neuroepithelium, ACE-2 has a higher expression in the supporting, basal and perivascular cells than in the olfactory neurons themselves. So, the olfactory disorders in post-COVID-19 tend to be primarily due to non-neural cellular changes.^12^

According to the British Rhinology Society Loss of Smell Consensus,^13^ olfactory training is recommended to patients with loss of smell longer than 2-weeks.

Olfactory training has been known to have a beneficial effect on the olfactory sense. The exposure to odors results in an increased growth of olfactory receptor neurons and an increased expression of olfactory receptors.^14^ It can explain the high evidence for recommending olfactory training after smell loss.

In despite we found a negative results to alpha-lipoic acid as an adjuvant in smell loss recover due to COVID-19, our results support the safety of olfactory training. This is a simple and relatively low-cost therapy that should be done by ENT or a general practitioner physician.^13^ A systematic review with meta-analysis also assessed the effectiveness and safety of OT for COVID-19 patients with smell disorders.^15^

This study has some limitations. Firstly, when we started this study, there were not any trial published about olfactory training or alpha-lipoic acid as a treatment to improve smell loss after COVID-19. So, sample size calculation was difficult. Thereat, we over expected a great size effect in alpha-lipoic acid use. Although this impacted on our study, the results confirmed the positive effect of OT for this condition.

Besides that, once traditional medical training overvalues drug intervention, complementary therapies cannot be forgotten. Also, our study did not have a control group where spontaneous recovery only was observed. We found an ethical issue in leaving part of participants without any promising treatment.

Finally, we did not assess the effects of alpha-acid lipoic and olfactory training in qualitative disorders, like parosmia. We planned to do that in another study. Furthermore, we plan to follow up these patients for 6–12 months after clinical onset to observe longer effects of treatment.

To our knowledge, the present study is the first double-blind, randomized trials to evaluate alpha lipoic acid as an adjuvant treatment for long-lasting severe olfactory disorders in COVID-19 patients. Given the high incidence of this infection in the population, there are significant and increasing numbers of patients with persistent smell disorders needing treatment.

## Conclusion

In the present study, the assessment of olfactory dysfunction based on smell test and VAS scores demonstrated no significant differences between the group that received alpha-lipoic acid associated to olfactory training and the group which received olfactory training with placebo pills. Although at the end of the intervention, anosmia was reported 2% in the intervention group versus 7.8% in the comparison group, no significant differences were found between groups. On the other hand, the change of smell test and VAS scores were significantly higher after the treatment in both groups. This reinforces the importance of olfactory training to recover smell loss due to COVID-19, especially the long-term ones. At last, we recommend further randomized clinical trials to reproduce these results.

## Funding

Brazilian Ministry of Science, Technology, and Innovation; the Brazilian National Council for Scientific and Technological Development (10.13039/501100003593CNPq).

## Conflicts of interest

The authors declare no conflicts of interest.
